# Molecular strategies for detecting chromosomal translocations in soft tissue tumors (Review)

**DOI:** 10.3892/ijmm.2014.1726

**Published:** 2014-04-04

**Authors:** MARGHERITA CERRONE, MONICA CANTILE, FRANCESCA COLLINA, LAURA MARRA, GIUSEPPINA LIGUORI, RENATO FRANCO, ANNAROSARIA DE CHIARA, GERARDO BOTTI

**Affiliations:** Pathology Unit, INT Pascale Foundation, I-80131 Naples, Italy

**Keywords:** soft tissue tumors, chromosomal translocations, molecular analyses

## Abstract

Approximately one third of soft tissue tumors are characterized by chromosomal aberrations, in particular, translocations and amplifications, which appear to be highly specific. The identification of fusion transcripts not only supports the diagnosis, but provides the basis for the development of novel therapeutic strategies aimed at blocking the aberrant activity of chimeric proteins. Molecular biology, and in particular, cytogenetic and qualitative and quantitative polymerase chain reaction technologies, allow with high efficiency and specificity, the determination of specific fusion transcripts resulting from chromosomal translocations, as well as the analysis of gene amplifications. In this review, various molecular techniques that allow the identification of translocations and consequent fusion transcripts generated are discussed in the broad spectrum of soft tissue tumors.

## 1. Introduction

Soft tissue sarcomas are a complex group of rare mesenchymal lesions, many of which are distinguishable from the others only through careful histological and ultramicroscopic investigations. Their diagnosis is problematic due to their rarity; 15–20% of these sarcomas are poorly differentiated, with a wide cellular variety that makes their classification difficult. In addition, histological subtypes which are morphologically similar present cytogenetic and molecular differences that influence the prognosis.

**Figure 1:**
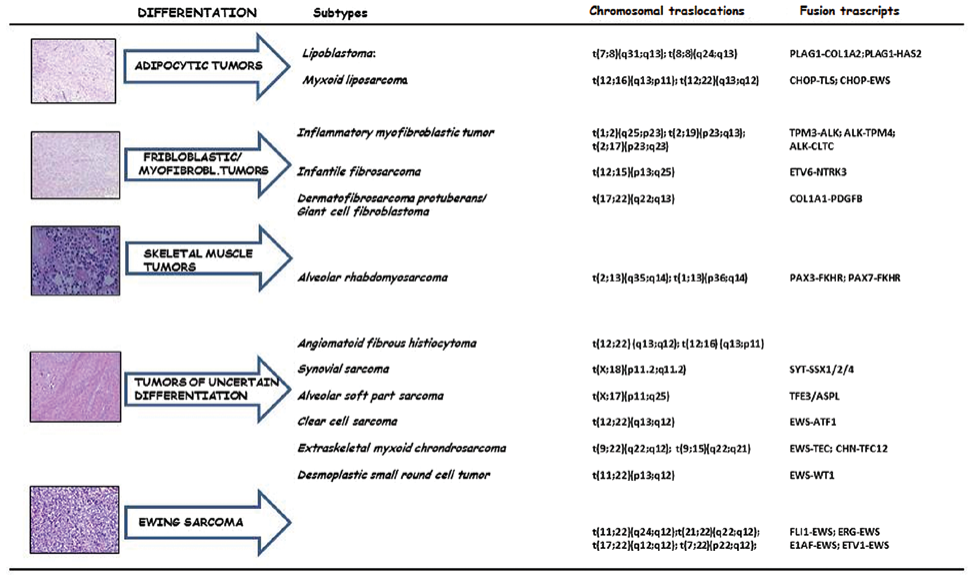
Schematic representation of soft tissue tumors, grouped on the basis of cell line differentiation, with chromosomal translocations and chimeric proteins produced.

Sarcomas are generally classified on the basis of tumor cell line differentiation rather than on the type of tissue from which they arise. A number of histotypes of differentiated tumor cells have been identified: adipocyte differentiation, fibroblast/myofibroblast differentiation, fibrohistiocytic differentiation, smooth/skeletal muscle differentation, tumors of uncertain differentation and a separate group which includes Ewing’s sarcoma (ES). However, not all histological types described present specific chromosomal alterations. For this reason, they are often grouped into ‘sarcomas with specific genetic alterations’ and ‘sarcomas with no specific genetic alterations’ (Fig. 1). In pleomorphic sarcomas, only cytogenetic and molecular biology allow a correct diagnosis, identifying specific chromosomal and molecular rearrangements (1).

**Figure 2:**
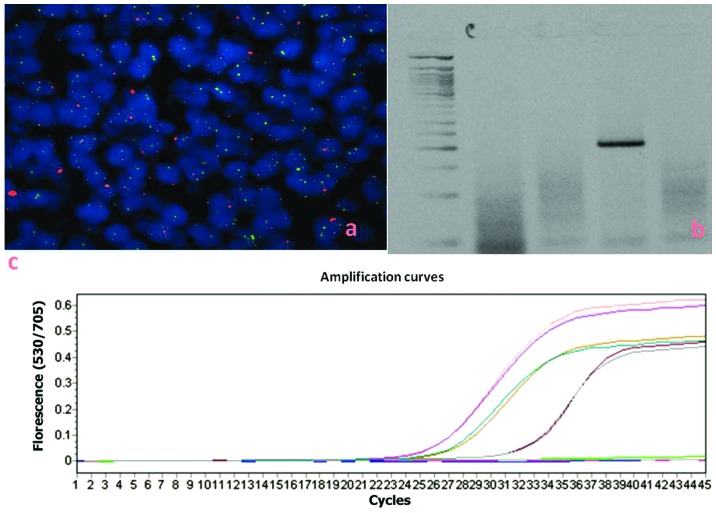
Different methods for gene translocation detection: (a) fluorescence *in situ* hybridization (FISH) (Vysis LSI CHOP Dual Color Break-apart Rearrangement Probe in a myxoid liposarcoma sample); (b) RT-PCR (fusion transcript of 195 bp in a myxoid liposarcoma sample); (c) real-time PCR (amplification curves associated to fusion transcripts in several myxoid liposarcoma samples).

Thus, the combination of morphological and molecular techniques represents an important progress, not only for a more adequate diagnostic definition, but also for the prognostic and therapeutic indications of these malignancies. Comparative genomics, *in situ* hybridization and gene array analysis allowed the rapid acquisition of the fundamentals of the biology/genetics of sarcomas (2). However, these techniques are not affordable for all laboratories. The cytogenetic techniques (Fig. 2a) and those associated with polymerase chain reaction (PCR) technology (Fig. 2b and c) have instead allowed us to extend the possibility to identify gene translocations/amplifications specific for these tumors in all diagnostic pathology laboratories, making the costs and methods more affordable for all.

In this review, we summarize not only all known chromosomal aberrations associated with soft tissue tumors, but also the different methods that help identify them and characterize the fusion transcripts produced.

## 2. Adipocytic tumors

Lipogenic tumors represent a heterogeneous group of lesions, mainly represented by liposarcomas. Among the principal histological subtypes of liposarcoma, myxoid liposarcoma (MLS) is the second most common, followed by well-differentiated liposarcoma (3), that have not been implicated in such chromosomal translocations or fusion genes.

### Lipoblastoma

Lipoblastomas are pediatric neoplasms, typically benign lesions, composed of adipose cells in different stages of maturation within a variably myxoid matrix, and they contain clonal rearrangements of chromosome band 8q12. In lipoblastomas, several chromosomal rearrangements have been described, involving the pleiomorphic adenoma gene 1 (PLAG1) oncogene. In particular, it was shown that the hyaluronic acid synthase 2 (HAS2) or collagen 1 α2 (COL1A2) gene promoter regions are fused to the entire PLAG1 coding sequence (4). The PLAG1 status was investigated through *in situ* hybridization techniques, particularly fluorescence detection [fluorescence *in situ* hybridization (FISH)] and chromogenic detection [chromogenic *in situ* hybridization (CISH)] (5).

### MLS

The non-random reciprocal translocation t(12;16)(q13;p11) is a characteristic of MLS (6,7). FISH is an alternative to ancestral cytogenetic methods, using painting probes against the centromeres of chromosome 12 metaphases and interphase nuclei (8–10). Early studies using FISH relied on the use of cosmid probes derived from YAC clones, which map at the CHOP locus (11,12). Currently, centralized laboratories specialized in FISH for the diagnosis of sarcomas, use dual-color, break-apart FISH probes spanning the genomic regions of FUS (16p11) (Vysis Inc., Downers Grove, IL, USA) (13,14).

Using Southern blot techniques, in samples with cytogenetic rearrangements in the region 12q13, it has been shown that CHOP/DDIT3 t(12;16)(q13;p11) is the gene involved in translocation (15). A chimeric transcript between the CHOP gene, which encodes a transcription factor, and the gene TLS/FUS that localizes in the region 16p11, is produced (16,17). The protein FUS/TLS interacts with several nuclear receptors and specific transcription factors. Through RT-PCR (18), different variants of the fusion transcript FUS/CHOP can be detected. The most frequent variants are the I and II variants, which are generated by alternative splicing between exon 2 of CHOP and exons 5 and 7 of the FUS gene (19–22). The different expression profile of the transcript FUS/CHOP has also been revealed by nested-PCR (23,24) or nested-PCR and direct sequencing (25,26) in frozen or paraffin-embedded biopsies. Cloning studies have demonstrated that the different variants have similar activities in transforming mesenchymal cells using the same molecular pathways (27).

Real-time PCR is a more specific and sensitive technique for both frozen and formalin-fixed, paraffin-embedded (FFPE) tissues (28). The TLS-CHOP chimeric product is also capable of promoting the development of MLS and tumorigenesis through the repression of the expression of a microRNA, miR-486, as demonstrated in studies on cloned cell lines from NIH3T3 fibroblasts and MLS tissues (29).

MLS can also present the t(12; 22)(q13,q12) aberration with its chimeric transcript EWS/CHOP fusion between exon 7 of the EWS gene and exon 2 of CHOP. FISH is an excellent method for detecting the presence of gene rearrangements in CHOP, but RT-PCR is the only method able to detect fusion partners, FUS or EWS (30,31). Patients who exhibit these fusion products show a more favorable clinical history compared to other patients positive for transcripts and different variants. Therefore, a correct diagnosis also related to biomolecular data is important, particularly in cases where the myxomatous change is minimal compared to their dominant counterparts which resemble pleomorphic malignant fibrous histiocytoma (32).

## 3. Fibroblastic/myofibroblastic tumors

Fibrosarcoma is a primary malignant tumor composed of immature fibroblasts. Several forms are recognized: infantile fibrosarcoma, which presents at birth or in early childhood, identical to the adult form apart from the clinical course and is much more favorable, as well as dermatofibrosarcoma protuberans (DFSP).

### Infantile fibrosarcoma

Infantile fibrosarcoma has the same characteristics of benign lesions of childhood, such as infantile fibromatosis and myofibromatosis (33). The specific translocation t(12;15)(p13;q25) (34), which makes the differential diagnosis possible, was identified by cytogenetic techniques and cloning of chromosome break sequences. FISH is a specific method, although more costly, and can be used to determine the status of chromosomal rearrangements of the regions 12p13 and 15q25 (35). Early studies were carried out using FISH probes or chromosome-specific bacterial artificial chromosome (BAC) clone probes (36). These were then replaced by commercial probes for dual-color fusion, the Ets variant 6 (ETV6) gene (Abbott Molecular, Inc., Des Plaines, IL, USA) (37).

The translocation generates a fusion transcript ETV6- neurotrophic tyrosine kinase, receptor, type 3 (NTRK3). The ETV6 gene is localized on chromosome arm 12p13, while the NTRK3 gene is in the chromosomal region 15q25 (38). Techniques of cloning and sequencing have shown that the fusion occurs between exon 5 of the ETV6 gene and exon 13 of the NTRK3 gene (38,39). Reverse transcriptase PCR (RT-PCR) is a specific method, fast and economical, for determining the presence of the fusion gene, ETV6-NTRK3, in fresh tissue samples or archived material (40–42).

### DFSP

DFSP is a rare variant that is derived from the fibrous component of the dermis and grows slowly, forming ulcers on the skin and subcutaneous tissues. The translocation t(17;22)(q22;q13) is highly specific for DFSP and can generate a chimeric transcript COL1A1/platelet-derived growth factor subunit B (PDGFB) and a supernumerary ring chromosome, r(17,22) (43,44).

Early studies on the detection of chromosomal translocations by FISH, were based on chromosome painting and α-satellite probes. Later studies reported the use of a dual-color dual-fusion BAC probe for COL1A1/PDGFB translocation, obtained by cloning vectors, BACs, covering the PDGFB gene entirely. In this manner, the different variants of the chimeric transcript were identified (45,46). Currently, commercial FISH probes are available, e.g., ZytoLight-SPEC COL1A1-PDGFB Dual Color Dual Fusion Probe (ZytoVision GmbH, Bremerhaven, Germany) (47). Different methods, such as Southern blot analysis, RT-PCR and FISH, based on frozen tissue specimens or using archival FFPE tumor samples have shown that COL1A1/PDGFB chimeric genes are present in all cases of DFSP (43,48,49).

Finally, to detect the copy number changes on the chromosomal regions, 17q and 22q, comparative genomic hybridization (CGH) studies have been performed, and have confirmed the amplification of 17q, but not always that of 22q (50,51)1. The breakpoint is at the level of exon 2 of PDGFB in the region 22q, but may involve several exons of the COL1A1 gene in the region 17q, such as exons 8, 10, 22, 24, 27, 32, 34, 38, 40, 45, 46 and 47, as shown by RT-PCR followed by sequencing analysis (52,53). The same methodology has allowed to identify additional variants of chimeric transcript, such as the one due to fusion at the level of exon 2 of PDGFB with COL1A1 gene but at the level of exon 41 (54). All possible variants of the fusion transcript can be detected using either multiplex RT-PCR (55) or FISH on paraffin-embedded tissues (46).

Real-time PCR provides a more sensitive alternative for analyzing the presence of the fusion transcript or the amplification of the regions affected by the rearrangement in fresh tissue or archived samples (56). Real-time PCR also allows the quantification of mRNA transcripts and the PDGFB chimeric gene, which is overexpressed compared to benign counterparts (normal tissue or dermatofibroma) (57,58).

## 4. Skeletal muscle tumors

Rhabdomyosarcomas originate most often in striated muscles at the level of the arms and legs and are more common in children than in adults. Three main forms are known: polymorphous rhabdomyosarcoma and alveolar rhabdomyosarcoma in adults, and embryonal rhabdomyosarcoma, which is more common in children. The tumor is ubiquitous and the most common sites are the arms, but also the head and neck, the urogenital tract and retroperitoneum. The progression is extremely aggressive, with a great tendency to recurrence and metastasis. The alveolar variant tends to have a worse prognosis than the embryonal variant.

### Alveolar rhabdomyosarcoma

Two chromosomal translocations, t(2;13)(q35;q14) and t(1;13)(p36;q14), are present in approximately 80% of all alveolar rhabdomyosarcoma cases (59–61).

The presence of t(2;13) in rhabdomyosarcoma cell lines was demonstrated by ancillary approaches, such as classical cytogenetics and FISH, using painting cosmid probes labeled with digoxigenin and biotin on both metaphases and interphase nuclei (62). t(2;13)(q35;q14) was firstly detected by FISH on interphase nuclei in minimally invasive biopsies of patients treated with neoadjuvant chemotherapy and then on non-suitable tumor material. In early hybridization studies, two cosmid clones were used in interphase cells with inserts of regions proximal or distal to the 13q14 breakpoint and a yeast clone with the inserted region distal to the 2q35 point (63). Present commercial dual-split-signal color FISH probes (Abbott Molecular) are more sensitive and specific to chimeric products, due to the two translocations involving the 13q14 region (64,65).

Studies on cDNA cloning and sequencing have shown that t(2;13)(q35;q14) produces a fusion transcript between the paired box 3 (PAX3) gene and FKHR gene, respectively (66,67); the PAX7-FKHR fusion transcript results from the t(1;13) translocation (68).

However, the survival and mortality rate in metastatic patients depend on the rearrangement type: the 4-year survival rate is 75% for patients with PAX7-FKHR vs. 8% for those with PAX3-FKHR. If PAX3-FKHR is expressed, there is a significant risk of death (P=0.019); besides, these patients may present bone marrow involvement (69).

The technical related issues associated with FISH in the diagnosis of alveolar rhabdomyosarcoma in children have been avoided by using RT-PCR, although this method is less sensitive than FISH (70,71); however, the chimeric transcript PAX3/FKHR has been detected in cell lines and in fresh tissue samples and FPPE samples using only very small amounts of tumor tissue (72,73). With multiplex RT-PCR it is also possible to calculate the residual disease (74) and allows the differential diagnosis of alveolar rhabdomyosarcoma and ES (72).

The presence of both translocation t(2;13)(q35;q14) and t(1;13)(p36;q14) products is detected with specific primers for the regions flanking the breakpoints in 13q and 1p, as well as for 2q (75), with RNA extracted from fresh or frozen tissue and formalin-fixed, paraffin-embedded tissue samples (76). The quality of the extracted RNA and the absence of specific primers for unusual variants are the technique limitations, but certainly the problems related to RNA quality will be reduced with fresh or frozen meterial or with the increase in the number of neoplastic cells after laser capture microdissection (77). The specificity of the test is very high (94–100%), compared to electrophoresis on an agarose gel. Discordant data have been obtained by analyzing the same samples by Southern blot analysis (78). A correct diagnosis also requires ancillary data, such as clinical history, immunohistochemistry and histology, while the uncertain cases may require further FISH investigation.

Multiplex fluorescent analysis of chromosomal translocations (MFACT) is another alternative method which can be used in place of conventional RT-PCR. This method has the advantage of completely eliminating the manipulation of the PCR products and thus it greatly reduces the risk of cross-contamination (79).

Real-time PCR using a hydrolysis probe, is a highly sensitive and specific method that reveals and further quantifies the chimeric transcripts PAX3-FKHR and PAX7-FKHR in the peripheral blood of patients, yielding similar results to nested-PCR. Patients positive for the fusion transcripts are at high risk of tumor progression (80). An initial screening for immunohistochemistry helps to select patients for molecular investigations, since it has been shown that only patients with alveolar rhabdomyosarcoma and not those with the embryonic phenotype that present with >50% of cells immunoreactive for myogenin, show the rearrangement of PAX (81).

## 5. Tumors of uncertain differentiation

Tumors that escape histological classification, as they lack a definite differentiation from a certain type of mesenchymal tissue, are encompassed in this category. They mostly occur between the ages of 15 and 40 years and are more common in males.

### Synovial sarcoma

Synovial sarcoma is the most common lesion in this group. The peak of incidence is the third decade of life, and the male/female incidence ratio is approximately 1.2:1. Synovial sarcoma can be mono- or biphasic and the difference is at the histopathological level: biphasic synovial sarcoma presents with epithelial and spindle cells, while monophasic synovial sarcoma almost always presents with spindle cells.

The t(x;18)(p11.2;q11.2) translocation has been observed in patients with this neoplasm, often as a unique cytogenetic abnormality (82), for which the SYT gene on chromosome 18 is juxtaposed to one of the two genes related, SSX1 or SSX2, but distinct on chromosome X (83,84).

The first cytogenetics and FISH studies on paraffin-embedded samples were performed using centromeric probes together with whole chromosome painting probes for chromosomes X and 18 (85). The translocation (X;18)(p11;q11) breakpoint has been demonstrated by both Southern blot analysis and FISH analysis using specific yeast artificial chromosome (YAC) probes (86,87). Dual-color break-apart probes, synthesized by cloning the breakpoint regions of interest in BAC clones and labeled with fluorescein-12-dUTP and TexRed-5-dUTP, were very useful in CISH and FISH (88). Currently, interphase FISH is performed on fixed, paraffin-embedded tissues using a commercially available LSI SS18 dual-color break-apart probe (Abbott Molecular/Vysis Inc.), a more specific and sensitive probe than non-commercial CISH probes (89,90).

The SS18-SSX chimeric product was detected even in FFPE samples by ISH using biotinylated tyramide and probes labeled with digoxigenin specific for the cDNA. The method produces signals in epithelial cells of biphasic sarcomas, with mild or focal positivity in monophasic tumors (91).

RT-PCR has a specificity of 100% with a sensitivity of 96% for the detection of the fusion transcript in paraffin-embedded lesions with the t(X;18) (SYT-SSX) translocation, limited only by the use of particular fixatives that produce a poor quality of extracted RNA (92,93). The molecular analysis evaluates the incidence of molecular variants, using sets of specific primers for SS18-SSX1 and SS18-SSX2; however, a statistically significant association between histological subtype (monophasic vs. biphasic) and SSX1 or SSX2 (94) has not been found. New variants for both SS18-SSX1 and SS18-SSX2 have been shown by sequencing the products of RT-PCR (95–97).

FISH and RT-PCR investigations are useful and necessary in those cases where the differential diagnosis between synovial and other spindle cell sarcomas (98) is difficult.

The fusion transcripts are detected and quantified by real-time RT-PCR, more sensitive and rapid than RT-PCR, using specific primers and TaqMan fluorescent probes complementary to the breakpoints in the genes involved in the translocation (99–101). Multiplex real-time PCR analyzes all variants of chimeric transcripts together, using appropriate sets of primers and probes: SS18-SSX1 has been shown to be present in both monophasic and biphasic synovial sarcomas more frequently than the SS18-SSX2 variant. They are mutually exclusive (102).

### Clear cell sarcoma (CCS)

CCS is a malignancy that can be morphologically confused with non-cutaneous melanoma as it presents the same immunophenotype. The differential diagnosis is possible thanks to the translocation t(12;22)(q13;q12), for which the chimeric gene EWSR1/ATF1 is formed in melanocytic tumors of soft tissues. Less commonly, CCS can be marked by t(2;22)(q34;q12), which produces the fusion transcript, EWSR1/CREB1, typical of gastrointestinal CCS, but that can also characterize CCS of soft tissue (103).

In order to characterize the translocation and its chimeric product, the first studies were conducted on cell lines, such as KAO, obtained from a girl of 9 years, or the HS-MM. melanoma cells, which were used as negative controls for t(12;22); CCS cells alone have been shown to be positive for the translocation and the EWS/ATF1 fusion gene, analyzed by FISH and RT-PCR, respectively (104).

Nowdays, slides of FPPE samples and tumor microarray (TMA) are routinely analyzed by interphase FISH with commercial probes LSY EWS dual-color break-apart (Vysis Inc.) and the positive cases can be analyzed by RT-PCR to determine the type of chimeric transcript EWS ATF1 (105,106). All cases of melanoma are negative to FISH for the same region, the 22q12 (107).

Four variants of the fusion transcript are detected by RT-PCR and sequencing, due to different breakpoints in the relevant gene regions: three subtypes are due to in-frame fusion and are type 1, 2 and 3, due to the fusion-EWS exon 8 and ATF1-exon 4, EWS-ATF1-exon 7 and 5, and EWS-ATF1-exon 10 and 5, respectively; the subtype 4 is due to the out-of-frame fusion of the region with exon 7 of EWS and exon 7 of ATF1. In addition to these four main transcripts, which may also occur together, there can be out-of-frame fusion between exon 10 of EWS and 3 of ATF1, or between exon 8 of EWS and 4 of ATF1, with insertion of nucleotides at the junction point (108,109). RT-PCR is performed using extracted RNA from either frozen or FFPE tissue (110).

Real-time PCR is much more sensitive than classical RT-PCR, and is highly specific and very helpful in the differential diagnosis of melanoma, on fresh or frozen or FPPE samples (111).

### Desmoplastic small round-cell tumor (DSRCT)

DSRCT is a rare and aggressive malignancy, with typical localization to serosa of the abdominal-pelvic peritoneum, with a male/female incidence ratio of 4:1 and occurs during adolescence or early adulthood. In 40% of patients, it metastasizes to the liver, lungs and lymph nodes.

The tumor shows epithelial and mesenchymal properties and neural differentiation, and the cells present the translocation t(11;22)(p13;q12), which juxtaposes the gene, EWSR1, to the tumor suppressor gene, WT1 (112,113), whose identification in specialist laboratories is very helpful for a correct differential diagnosis, complicated by similarities with other small round cell tumors. The fusion protein EWSR1/WT1 acts as a potent transcriptional activator (114).

In clinical diagnostics of FFPE tissue sections, interphase FISH is routinely performed with a commercially available EWSR1 (22q12) dual color, break-apart rearrangement probe, but the t(11;22) is also found in 90% of EWS/primitive neuroectodermal tumor (PNET) and CCS cases (105).

The biological differences between DSRCT, ES and CCS can be explained by the presence of the different partners in the EWS gene translocation. Studies using Southern blot analysis, multi-enzymatic digestion and northern blot analysis have demonstrated that gene rearrangement in the region 22q12 produces the fusion of the EWS gene on 11p13 with WT1, the gene involved in Wilms tumor. RT-PCR, with the use of a primer for exon 7 of EWS and primers for exons 8 or 9 of WT1, confirmed the data (115). The chimeric mRNA is due to an in-frame fusion of the amino-terminal domain of EWS with the zinc-finger DNA-binding domain of WT1, which can undergo alternative splicing (116). Chromosomal translocation and fusion with EWS affect two independent biochemical functions of WT1, binding activity to DNA and transcriptional regulation, a deregulation that influences tumorigenesis in intra-abdominal DSRCT (114,117).

The variability in the breakpoint EWS produces molecular variants of the fusion gene EWS-WT1, as happens for the chimeric gene EWS-FLI1 in ES, such as an in-frame splicing of exon 9 of EWS to exon 8 of WT1, a variant found in a DSRCT unusually arising on hand, or an in-frame junction of EWS to exons 8–10 of WT1 (118).

To differentiate DSRCT from EWS/PNET, when the genetic information is not available, immunohistochemistry is recommended with an anti-WT1 antibody, highly specific and sensitive, that is a reliable index for the presence of the EWS-WT1 chimeric product (119).

RT-PCR can detect all chimeric messages that are formed by fusion between exons 1–7 of EWS and exons 8–10 of WT1 (120). Multiple in-frame cDNA can be also detected. It is produced by large internal deletions, insertions of small parts of heterologous DNA at the site of the junction between the two exons EWS and WT1, or the loss of exon 6 of EWS or exon 9 of WT1. The molecular diversity and functionality of these fusion transcripts may have significant biological implications for their tumorigenic potential (121).

### Inflammatory myofibroblastic tumor (IMT)

IMT is a mesenchymal tumor that presents with fibroblastic and myofibroblastic spindle cell proliferation mixed with lymphocytes, plasma cells and histiocytes (122). It can commonly occur in children, teenagers and adults under the age of 40. It was described for the first time in lungs and remains the most frequent mesenchymal endobronchial tumor in childhood (123). IMT can localize in any anatomical site, but it rarerly occurs in the liver. The lesion often presents with ambiguous morphological, structural and vascular properties, since it has both an inflammatory and neoplastic nature and therefore, diagnosis can be difficult.

Cytogenetic analysis has revealed clonal chromosomal abnormalities exhibiting the neoplastic nature of the disease. Approximately half of inflammatory myofibroblastic tumors present rearrangements of the locus of the anaplastic lymphoma kinase (ALK) gene on chromosome 2p23, resulting in the aberrant expression of ALK. FISH of interphase nuclei of FPPE samples were carried out using a commercial dual-color (red and green) ALK probe (Vysis Inc.), that labeled at telomeric region in SpectrumOrange and at centromeric region in SpectrumGreen of chromosome 2 (124,125). FISH leads to a correct diagnosis of IMT, even in cases in which the inflammatory component is minimal and during prenatal life as well (126). It is also possible to perform FISH on samples of fine needle aspiration (FNA) or endoscopic ultrasound-guided FNA (EUS-FNA) (127,128).

The disease can also present morphological characteristics of other similar lesions, such as child congenital fibrosarcoma (CIFS) and hemangiopericytoma, but the identification of the present transcript fusion by RT-PCR facilitates the correct diagnosis (129,130).

ALK, which is normally downregulated in neural tissues, is overexpressed in IMT cells with the 2p23 rearrangement, in which the N-terminal domain of tropomyosin (TPM) is fused to the C-terminus of ALK. Cloning studies have shown two fusion products, TPM4-ALK and TPM3-ALK, which encode for oncoproteins with constitutive kinase activity (131,132). RT-PCR seems the best method to identify the ALK fusion transcripts (133,134).

ALK can be also fused with clathrin heavy chain (CTLC), a gene localized to 17q23; however, patients that are t(2;17) positive show other abnormal karyotypes as well (135).

RT-PCR with specific pairs of primers and direct sequencing of the amplification products have also enabled the identification of new partners of ALK, such as dynactin-1, when the alteration der(2) t(2;12)(p23;q11) is present (136), or when the SEC31L1/ALK fusion gene, due to translocation t(2;4)(p23;q21), is present in two variants of different lengths (137).

A partial response by the inhibitor of ALK, crizotinib, has been reported in a patient with inflammatory myofibroblastic tumor with ALK translocation (138,139).

### Alveolar soft-part sarcoma (ASPS)

ASPS represents approximately 0.5–1% of soft tissue sarcomas and typically develops in adolescents and young adults; in children the localization is typical on the head and neck (140,141). Although growth is indolent, up to 79% of patients develop metastatic disease, since a significant percentage of them is resistant to conventional chemotherapeutic drugs. The development of chemoresistant metastases contributes to the increase in the mortality rate.

The translocation der(17)t(X;17)(p11;25) is the ASPS biomolecular marker (142) that causes the fusion of the transcription factor, TFE3, on Xp11.22, with a novel gene on 17q25, termed ASPL (ASPSCR1). The chimeric product, ASPL-TFE3, acts as a transcription factor and induces an aberrant transcription of genes regulated by TFE3 (143,144).

The translocation is confirmed by dual-and triple-color FISH on metaphases and interphase nuclei (145). Early studies of fluorescence *in situ* were performed using YAC and cosmid probes from the genomic regions of interest (146).

RT-PCR can be performed on frozen and FPPE tumor tissue to detect the presence of the resulting ASPSCR1-TFE3 fusion transcripts and its variants. The location of the fusion transcript, if present, leads to the proper diagnosis of ASPS, previously considered as a subgroup of RCC in children (147,148). The chimeric products of t(X;17)(p11;25) are detected by nested RT-PCR, which is more sensitive, also in circulating tumor cells in peripheral blood of ASPS patients with distant metastases (149).

Preliminary clinical studies have shown that patients with ASPS positive for der(17)t(X;17)(p11;25) respond to treatment with trabectedin, the only currently available clinical drug which has shown to be effective in the treatment of this disease (150).

### Extraosseous myxoid chondrosarcoma (EMC)

EMC presents strings of small acidophilic cells similar to chondroblasts in a myxoid stroma, and occurs particularly in the lower extremities, particularly in the fifth decade of life with a male/female incidence ratio of 2:1 (151). Patients may have long-term survival; however, local recurrences and metastases occur in approximately half of the cases, commonly in the lungs (152–154). Unlike bone chondrosarcoma, EMC behaves in a less aggressive manner. Therefore, it is deemed as two prognostically distinct entities (152).

Previous cytogenetic data have included a translocation t(9;22)(q22–31;q12), that produced the EWS/CHN chimeric gene and showed complex karyotypes (155,156). The translocation t(9;17)(q22;q11.2) is less frequent and combines with the CHN RBP56 gene, also known as TAF15, TAF2N or TAFII68 (157). A third translocation was also identified, typical of EMC, but less common, affecting chromosomes 9 and 15 and forming the chimeric gene, CHN/TCF12. Gene TCF12, also known as HTF4, presents different isoforms by alternative splicing and the breakpoint affects the region of intron 5 (158,159). Further molecular analyses have revealed additional chromosomal aberrations that can aid in the diagnosis of EMC, identifying other chimeric variants, such as the fused trascript *TFG* (TRK-fused gene)/CHN associated with t(3;9)(q11-q12;q22) (156,160,161).

The translocation occurs due to different breakpoints in various introns of the gene, EWS and CHN (termed NR4A3, NOR1 or TEC) (162), resulting in different variants of the chimeric products, EWS/CHN. The most frequent are: type 1, for the fusion between exons 12 of EWS and 3 of CHN, and type 5, between exons 13 of EWS and 3 of CHN. The chimeric gene RBP56/CHN is always formed by fusion between exons 6 of RBP56 and 3 of CHN. The mapping of the different regions of breakpoints in the EWS and CHN genes has shown that there are no sequence-specific recombinases or homology to explain the various breakpoints, due to other associated events such as deletions, duplications and inversions (163).

In FISH on formalin-fixed, FNA biopsy and paraffin-embedded tissues, using commercial LSY EWSR1 (22q12) dual-color, break-apart probe (Vysis Inc.), it is possible to demonstrate the presence of the EWSR1 gene rearrangement (13,164,165). The translocation at gene NR4A3 has been shown by dual-color FISH using a custom probe, synthesized by means of BAC and telomeric chromosome clones, labeled with SpectrumGreen and SpectrumOrange (166). Variant translocations were also detected by interphase FISH, such as t(9;15)(q22;q21) and t(7;9;17)(q32;q22;q11), with satellite probes for chromosomes 7, 8 and 12, and telomeric probes for 1q and 19q (Vysis Inc.) (167).

RT-PCR on archival FPPE samples, using specific pairs of primers, is a useful technique for detecting both the chimeric products due to the main translocations, such as EWS-CHN or RBP56-CHN (168), and different transcripts from EWSR1/TAF15/TFG-NR4A3 fusion, such as EWSR1-CREB1 fusion transcript which is present in cases of primary pulmonary myxoid sarcoma (169).

## 6. Ewing’s sarcoma

ES is a bone cancer, the most frequent after osteosarcoma, histologically characterized by sheets of small round cells, blue staining with H&E, which can be confused with lymphoma or embryonic rhabdomyosarcoma. It is rare in newborns and after the age of 30, with a higher incidence at the age of 16. ES and PNET not only have similar microscopic characteristics, but also show the same genetic alteration, a translocation (170–172); thus, they are subsequently grouped in a class of tumors defined as ‘Ewing’s sarcoma family tumors’ (ESFT). The typical translocation affects the region of chromosome 22 in which the family of ETS transcription factors are mapped. In 90% of cases the chimeric gene has the region of chromosome 11 as a partner of the gene EWS, producing the fusion transcript, EWSR1-FLI1; chromosome 21 is the less frequent partner and in particular the translocation forms the product gene, EWSR1-ERG (173). The region of chromosome 22 with EWSR1 can translocate into other chromosomal regions, t(21;22), t(7;22), t(17;22) and t(2;22), producing different chimeric transcripts according to the fusion partner (ERG, ETV1, E1AF and FEV) (173–175).

Colorimetric or fluorescence studies have used YAC probes, tested on paraffin-embedded tissue sections (176), while others have used and validated constructed probes on cell lines (177). Using a triple-target FISH on interphase nuclei, metaphase chromosomes and DNA fibers, it has been proven how the transcript EWS/ERG, in particular, may occur. The technique has been performed with co-hybridization of probes cloned in cosmids, complementary to the telomeric and centromeric regions of the region with the EWS breakpoint. FISH, in particular, has shown that an inversion of the ERG gene or part of it may be followed by an insertion in the EWS gene on der(22) (178). It is now of routine use to investigate translocations involving the EWSR1 gene using a dual interphase LSY color break-apart EWS-FISH (179), while the different variations of the formed fusion transcripts have been investigated by RT-PCR with specific pairs of primers (180).

RT-PCR amplifies RNAs extracted from fresh or cryopreserved tissue samples; however, the results are specific and confirmed on archivial FPPE material (181). The method is also sensitive to detect minimal residual disease (182) and a simultaneous detection of all chimeric products can be done using a mixture of primers. This method can be very useful in clinical practice, to guarantee diagnosis, to perform investigations of minimal metastatic and residual disease and to evaluate the prognostic significance of the subtypes of chimeric transcripts even when fresh tumor tissue is not available (182).

The specificity of EWS transcripts with their respective partners for ES was tested using nested RT-PCR on different samples of FPPE tissue (183,184).

Combining the biomolecular investigations by nested RT-PCR, more sensitive than conventional RT-PCR, with cytogenetic analysis by FISH in FPPE samples, a clear diagnosis of ES/PNET is possible. In particular, *in situ* hybridization of nuclear extraction (NE-FISH) is more reliable than that of thin-section (TS-FISH) in detecting the translocation of EWSR1 (185). The specificity of the amplification product for nested RT-PCR is confirmed by the subsequent digestion of the PCR fragments with different restriction endonucleases, a rapid method to determine the combination of exons present in a chimeric mRNA (186).

The use of western blotting to detect the fusion protein of 68-kDa EWS/FLI1 in samples of surgical biopsies and in fine needle aspirates of ES, also detected in cell lines of ES, bypasses the problems related to the quality of mRNA extracted from paraffin-embedded samples or the risk of contamination in amplification techniques, such as RT-PCR (187).

It is possible to genotype the allelic discrimination for single nucleotide polymorphisms (SNPs) in the EWS gene using a TaqMan assay real-time-PCR. The analysis revealed a higher incidence of the presence of homozygous TT in patients with ES. The analysis also allowed the detection and identified the region around the SNP, formed by a hexamer palindrome (5′-GCTAGC-3′) and three nucleotides (GTC), very close to the breakpoints in both the EWS and FLI1 genes. In patients homozygous for this set of alleles, there a tendency to fracture doubles, increasing the possibility of a translocation. SNP can be then a candidate marker for susceptibility to ES (188). Moreover, investigations by TaqMan real-time-PCR with a set of pairs of specific primers and probes can quantify the different chimeric transcripts in ES (EWS-FLI1, EWS-ERG, EWS-TV1, EWS-ETV4 and EWS-FEV) (189).

## 7. Conclusion

A number of studies have been undertaken to increase knowledge on chromosomal aberrations and facilitate the diagnosis of subsequent lesions, detecting specific translocations and chimeric products (190–192). The described chromosomal rearrangements not only aid in the diagnosis and classification of soft tissue tumors, but are particularly useful in the differential diagnosis of patients with an uncertain or dubious morphology (193). Routine techniques, such as FISH and RT-PCR, can be within the reach of pathology laboratories, helping the pathologist in the diagnosis of such neoplasms (194). Obviously, as for all biomolecular methods, an essential condition is mandatory for the correct development of these methods and to ensure useful results for diagnosis; this includes all pre-analytical stages of preparation of the biological sample. Appropriate sampling and all stages, ranging from fixation/inclusion to the cutting of sections destined for FISH and purification of nucleic acids, must be conducted in the correct manner and by established standardized procedures.
